# "Confessio Medici"

**Published:** 1908-05-16

**Authors:** 


### "Confessio Medici."

It is rare to find a good volume of essays among the swarms of new books issuing daily from the publishers' shops The modern essayist seldom gains popularity, and as seldom deserves it; for the writing of essays is an art which can only be mastered after long practice by those who are endowed with special qualities of mind and spirit. Few nowadays have that distinction and flexibility of style, that vigour of expression and variety of treatment, which characterised the great essayists of the past. It was, therefore, with some misgivings that we learnt of the appearance of a collection of essays, whose title challenged comparison with the "Religio Medici" of Sir Thomas Browne. But the first few pages showed the folly of prejudgment. "Confessio Medici" is the work of a skilled writer, and can stand on its own merits without fear of damaging contrasts. A member of our profession has won success in a difficult field of literature; and we are proud to think that medical practice, which supplies him with his theme, will gain in dignity and public esteem from its treatment in his book. "Some Bookes are to be Tasted . . . . and Some Few to be Chewed and Digested." This volume will repay either mode of perusal, but especially will it repay the longer method. The essays are pictures of medical life, drawn with a sure and sympathetic hand, and through them we see always the point of view of a cultured, philosophical mind. The preface explains the title: "Confessio" is not a confession of sins or a betrayal of secrets. "I only want to confess what I have learned, so far as I have come, from my life, so far as it has gone." These are the author's words, and they strike the key-note of the book.

The temptation to quote further from these essays is great: yet we have not space even to point out the passages which pleased us most. A connecting thread runs through the book, and each part is in its right place in relation to the whole. The pictures of hospital life and of practice are vivid, but never theatrical. "Truth to the fact and a good spirit in the treatment"—to use the words of one whose influence can be traced in the author's style—are guiding principles never out of sight. At the hands of an artist less refined in thought, less scholarly and adroit in manner, these everyday scenes of medical life might well have sunk to the level of commonplace. The author is no materialist: he knows life at first hand, he understands human nature, he has imaginative force, and he believes in Psyche. But he sternly commands us to hold fast to material things in the pursuit of our art—or else woe betide us and our patients. Philosophy must be kept for odd moments, away from the bedside; culture is inimical to the spirit of practice, and must be excluded from the furniture of the doctor's mind. And yet every sentence of the book bears the imprint of wide reading and of a delicate sense of the precise value of words, and seems to mock the writer's professional distrust of culture, so often expressed.

Of the various essays, "Wreaths and Crosses" is perhaps the best from a literary point of view; but all are good. To the medical reader the essay on "Retirement" will especially appeal. The story of the sudden enforced inactivity of Velox, the successful consultant-practitioner in a Midland city, is painfully life-like. So is the sketch of the slow dissolution of Prudens, the eminent surgeon, whom few will fail to recognise. The contrast between these characters and those who may be said to have been retiring all their lives is excellently well drawn. Indeed, throughout the book, the author reflects the spirit and the body of practice as they really are. "Confessio Medici" cannot fail to provide pleasure and material for thought to all those who belong to our profession, and to many others who are interested in the medical life from within, and who can enjoy good literature.

